# Intraoperative hypotension is associated with increased postoperative complications in patients undergoing surgery for pheochromocytoma-paraganglioma: a retrospective cohort study

**DOI:** 10.1186/s12871-020-01066-y

**Published:** 2020-06-12

**Authors:** Nan Li, Hao Kong, Shuang-Ling Li, Sai-Nan Zhu, Zheng Zhang, Dong-Xin Wang

**Affiliations:** 1 Department of Anesthesiology and Critical Care Medicine, Peking University First Hospital, No.8 Xishiku street, Beijing, 100034 China; 2 Department of Biostatistics, Peking University First Hospital, Beijing, China; 3 Department of Urology, Peking University First Hospital, Beijing, China

**Keywords:** Pheochromocytoma-paraganglioma, Intraoperative hypotension, Postoperative complications

## Abstract

**Background:**

Dramatic hemodynamic fluctuation occurs frequently during surgery for pheochromocytoma or paraganglioma. However, the criteria of intraoperative hemodynamic instability vary widely, and most of them were defined arbitrarily but not according to patients’ prognosis. The objective was to analyze the relationship between different thresholds and durations of intraoperative hyper−/hypotension and the risk of postoperative complications in patients undergoing surgery for pheochromocytoma or paraganglioma.

**Methods:**

This was a retrospective single-center cohort study performed in a tertiary care hospital from January 1, 2005 to December 31, 2017. Three hundred twenty-seven patients who underwent surgery for pheochromocytoma or paraganglioma, of which the diagnoses were confirmed by postoperative pathologic examination, were enrolled. Those who were less than 18 years, underwent surgery involving non-tumor organs, or had incomplete data were excluded. The primary endpoint was a composite of the occurrence of AKI or other complications during hospital stay after surgery. Multivariate Logistic regression models were used to analyze the association between different thresholds and durations of intraoperative hyper−/hypotension and the development of postoperative complications.

**Results:**

Forty three (13.1%) patients developed complications during hospital stay after surgery. After adjusting for confounding factors, intraoperative hypotension, defined as systolic blood pressure (SBP) of ≤95 mmHg for ≥20 min (OR 3.211; 99% CI 1.081–9.536; *P* = 0.006), SBP of ≤90 mmHg for ≥20 min (OR 3.680; 98.8% CI 1.107–12.240; *P* = 0.006), SBP of ≤85 mmHg for ≥10 min (OR 3.975; 98.3% CI 1.321–11.961; *P* = 0.003), and SBP of ≤80 mmHg for ≥1 min (OR 3.465; 95% CI 1.484–8.093; *P* = 0.004), were associated with an increased risk of postoperative complications. On the other hand, intraoperative hypertension was not significantly associated with the development of postoperative complications.

**Conclusions:**

For patients undergoing surgery for pheochromocytoma or paraganglioma, intraoperative hypotension is associated with increased postoperative complications; and the harmful effects are level- and duration-dependent. The effects of intraoperative hypertension need to be studied further.

## Background

Pheochromocytoma and paraganglioma are uncommon neuroendocrine diseases with a combined annual incidence rate from 0.29 to 0.46 per 100,000 person-years [1]. Surgical resection is the mainstay for the management of these tumors. Because of excessive intraoperative catecholamine release, especially during endotracheal intubation, creation of pneumoperitoneum, manipulation of tumor, and dividing adrenal vein [2–4], dramatic hemodynamic fluctuation occurs frequently, making the perioperative management a great challenge for the anesthesiologists and intensivists.

Much attention has been paid to the hemodynamic management of these patients during surgery [5–7]. However, the criteria of intraoperative hemodynamic instability vary widely, including absolute thresholds and/or relative changes of systolic blood pressure (SBP), mean arterial pressure (MAP) and even heart rate (HR), leading to reported rates range from 22.7 to 91.1% [8–10]. More importantly, most of these criteria were defined arbitrarily but not according to the risks of postoperative morbidity and mortality.

In previous studies of patients following non-cardiac surgery, intraoperative hypotension is found to be associated with increased risks of acute kidney injury (AKI) [11, 12], myocardial injury [12, 13], delirium [14], stroke [15], and even death [16, 17]. On the other hand, intraoperative hypertension is also reported to be associated with adverse outcomes [18]; although there are evidences that the harmful effects of intraoperative hypertension might be less than those of intraoperative hypotension [19]. Extreme hyper- and hypotension are more likely to occur during surgery for pheochromocytoma or paraganglioma. We, therefore, hypothesized that intraoperative hyper- and/or hypotension were associated with the development of postoperative complications.

In this study, we aimed to analyze the relationship between different thresholds and durations of intraoperative hyper−/hypotension and the risk of postoperative complications in patients undergoing surgery for pheochromocytoma or paraganglioma.

## Methods

This was a retrospective single-center cohort study. The database used in the present study was established years ago after approval by the Clinical Research Ethics Committee of Peking University First Hospital, Beijing, China (2016–1062) [10]. For the purpose of the present study, we updated the database after another ethical approval (2018–47). All data were retrospectively collected. Because of the retrospective nature and that all data of patients were collected from the medical records, the local Ethics Committee agreed to exempt the written informed consent. However, the privacy of all participants was strictly protected.

### Patients

Potential participants were patients who underwent surgery for pheochromocytoma or paraganglioma, of which the diagnoses were confirmed by postoperative pathologic examination, from January 1, 2005 to December 31, 2017 in Peking University First Hospital. Patients who met any of the following criteria were excluded: (1) age less than 18 years; (2) surgery involving non-tumor organs; (3) incomplete data collected from the medical record system.

### Data collection

Patients’ data were collected from the electronic medical record system of the hospital. Baseline and preoperative data included demographic characteristics (age, gender, body mass index [BMI]), previous comorbidity, American Society of Anesthesiology (ASA) classification, preoperative hemoglobin, baseline serum catecholamine level, size and location of tumor, medical treatment, as well as blood pressure and heart rate before surgery.

Intraoperative data included period of surgery, type and duration of anesthesia, type and duration of surgery, estimated blood loss, minimal hemoglobin, blood transfusion, positive fluid balance, hemodynamic parameters, use of antihypertensive drugs, and use of vasopressors.

Postoperative data included use and duration of vasopressors, intensive care unit (ICU) admission, use and duration of mechanical ventilation, length of stay in ICU and hospital after surgery, occurrence of complications during hospital stay, and in-hospital mortality.

The primary endpoint was a composite of the occurrence of AKI or other complications during hospital stay after surgery. AKI and its stage were diagnosed according to the Kidney Disease Improving Global Outcomes (KDIGO) criteria using the maximal change in serum creatinine value compared with the preoperative baseline and urine output [20]. Other postoperative complications were defined as any deviation from the normal postoperative course which required pharmacological treatment or interventional procedures, i.e., grade II or higher on the Clavien-Dindo classification [21].

To ensure the accuracy of the outcome database, two investigators collected the information of postoperative outcomes separately and independently. They were blinded to intraoperative blood pressure management at the time of postoperative data collection. In case of any difference between the two investigators, final agreement was achieved by rechecking medical records and full discussion with a senior physician.

### Preoperative antihypertensive treatment

For a patient with diagnosed or strongly suspected pheochromocytoma or paraganglioma, the attending physician/surgeon would initiate the α-blocker therapy with a starting dose while decreasing or ceasing other antihypertensive drugs used before. The dose of α-blockers was gradually adjusted to achieve a target blood pressure while avoiding orthostatic hypotension. During this process, a calcium channel blocker and/or a β-adrenergic receptor blocker might be added when considered necessary.

### Intraoperative blood pressure management

In case of hypertension which commonly occurred during stimulation or tumor manipulation, anesthesia depth was firstly checked and deepened when necessary. When hypertension persisted, intravenous antihypertensives such as phentolamine, urapidil and/or nicardipine as well as a short-acting beta-blocker esmolol were administered. Intravenous antihypertensives were ceased shortly before ligation of tumor vessels. In case of hypotension which frequently occurred after tumor removal, intravascular volume was re-evaluated and fluid resuscitation was performed when considered necessary. When hypotension persisted, intravenous vasopressors such as phenylephrine, norepinephrine and/or epinephrine were administered to maintain adequate organ perfusion.

### Processing of intraoperative blood pressure data

The data of blood pressure was captured every 10 s and stored by the electronic anesthesia information system in a real-time manner and were examined separately and independently by two investigators (NL and HK). During episodes when no blood pressure was recorded or when artifacts were marked by the anesthesiologists, the last blood pressure was used to replace the missing data.

For the purpose of analysis, absolute thresholds of SBP with different durations were used to define intraoperative hyper−/hypotension. We adopt this criterion because SBP is the primary target of intervention [22]. Specifically, SBPs of ≥160, 180 and 200 mmHg were used as thresholds for intraoperative hypertension and those of ≤100, 95, 90, 85, and 80 mmHg for intraoperative hypotension, with durations of 1, 5, 10, 20, 30, 40 and 50 min, respectively.

### Statistical analysis

Patients’ data were analyzed according to the development of postoperative complications or not. Numeric data with normal distribution were compared using independent samples t rest; numeric data with non-normal distribution or ranked data were compared using Mann-Whitney U test. Categorical data were compared using chi-square test or Fisher’s exact test. Time-to-event data were analyzed by Kaplan-Meier estimator, with difference between groups compared by log- rank test. The association between different thresholds and durations of intraoperative SBP and the development of postoperative complications were analyzed using the Logistic regression models. Independent variables with *P* values < 0.10 in univariate analyses or were considered clinically important were included in the multivariate models to adjust for confounding factors. Two-sided *P* values of < 0.05 were considered as statistically significant. For multiple comparisons, the thresholds of *P* values were adjusted with Bonferroni correction. Statistical analyses were performed with the SPSS statistical package version 25.0 (IBM SPSS Inc., Chicago, IL, USA).

## Results

### Patients

**Fig. 1:**
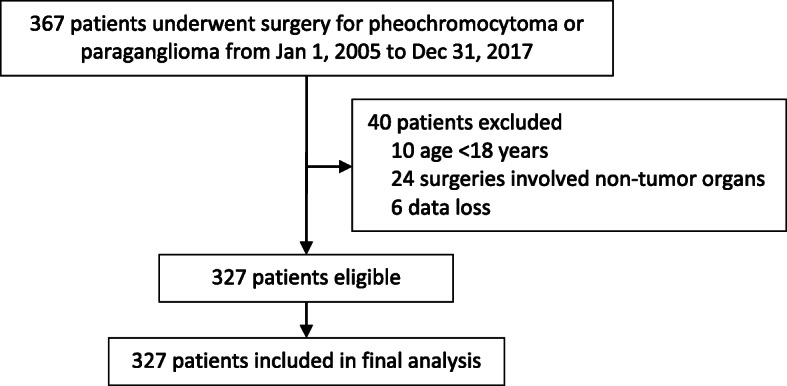
Flowchart of the study

From January 1, 2005 to December 31, 2017, 367 patients underwent surgery for pheochromocytoma or paraganglioma; among these, 327 met the inclusion/exclusion criteria and were included in final analysis (Fig. 1).

Of the enrolled patients, 43 (13.1%) developed AKI or other complications during hospital stay after surgery (Table 1). When compared with patients who did not develop postoperative complications, those who did had a higher male ratio, more comorbidity of diabetes mellitus and previous stroke, larger tumor diameters, and more paragangliomas; they received more calcium channel blockers and combined antihypertensive therapies, had higher preoperative BP and HR, and underwent more surgery between 2010 and 2013 (Table 2). During surgery, patients who developed postoperative complications underwent longer anesthesia/surgery and more open surgery, lost more blood, had lower hemoglobin level, received more blood transfusion, had more positive fluid balance, and were given more antihypertensives and vasopressors (Table 2).

**Table 1:** Occurrence and severity of individual complications following surgery

Complications	Total number	Severity of postoperative complications ^*a*^					
		II	IIIa	IIIb	IVa	IVb	V
Stroke ^*b*^	2	2	–	–	–	–	–
Respiratory complications	6	4	2	–	–	–	–
Pulmonary infection ^*c*^	4	4	–	–	–	–	–
Pleural effusion ^*d*^	2	–	2	–	–	–	–
Cardiovascular complications	3	2	1	–	–	–	–
New onset arrhythmia ^*e*^	1	–	1	–	–	–	–
Acute myocardial infarction ^*f*^	2	2	–	–	–	–	–
Surgery-related complications	6	3	–	1	1	1	–
Surgical bleeding ^*g*^	3	–	–	1	1	1	–
Ileus ^*h*^	3	3	–	–	–	–	–
Thromboembolic complications	3	2	–	–	1	–	–
Pulmonary embolism ^*i*^	1	–	–	–	1	–	–
Deep venous thrombosis ^*j*^	2	2	–	–	–	–	–
Hypoglycemia ^*k*^	9	9	–	–	–	–	–
Urinary tract infection ^*l*^	1	1	–	–	–	–	–

Data are number

^*a*^ According to Clavien-Dindo classification

^*b*^ Persisted new focal neurologic deficit and confirmed by neurologic imaging

^*c*^ Presence of at least one of the following manifestations, i.e., increased or color-changed sputum, new or changed pulmonary infiltrates, fever, and leukocyte count > 12,000/mm^3^, and required antibiotic therapy

^*d*^ Confirmed by chest X-ray or ultrasound examination and required drainage, aspiration, and/or diuresis after albumin administration

^*e*^ New onset atrial fibrillation or paroxysmal supraventricular tachycardia that necessitated antiarrhythmic therapy

^*f*^ Concentration of cardiac troponin I exceed the diagnostic criteria for myocardial infarction as well as new Q waves (lasts for 0.03 s) or continuous (4 days) abnormal ST-T segment

^*g*^ Bleeding after surgery that required secondary surgical hemostasis

^*h*^ Lack of bowel movement, flatulence, and requirement of parenteral nutrition for more than 1 week after surgery

^*i*^ Confirmed by computed tomographic pulmonary angiography

^*j*^ Confirmed by deep venous ultrasonography

^*k*^ Defined as a documented serum blood glucose level of less than 55 mg/dL

^*l*^ Confirmed by urinalysis and urine culture and necessitated antibiotic therapy

**Table 2:** Baseline and intraoperative variables

Variables	Without postoperative complications (*n* = 284)	With postoperative complications (*n* = 43)	*P* value ^*a*^
Age (years)	46 ± 15	48 ± 16	0.379
Male gender ^*b*^	119 (41.9%)	28 (65.1%)	**0.004**
BMI (kg/m^2^)	23.3 ± 3.5	23.2 ± 3.7	0.811
Preoperative comorbidity			
Diabetes mellitus	38 (13.4%)	12 (27.9%)	**0.014**
Coronary heart disease	18 (6.3%)	1 (2.3%)	0.487
Previous stroke	9 (3.2%)	7 (16.3%)	**0.002**
ASA classification ^*b*^			0.223
1	14 (4.9%)	2 (4.7%)	
2	210 (73.9%)	26 (60.5%)	
3	59 (20.8%)	15 (34.9%)	
4	1 (0.4%)	0 (0.0%)	
Preoperative examination			
Hemoglobin (g/L)	134 ± 18	133 ± 24	0.851
Serum catecholamine			
Dopamine (pmol/L)	0.10 (0.05 to 0.31)	0.10 (0.06 to 0.44)	0.412
Norepinephrine (pmol/L)	11.7 (4.3 to 26.7)	15.3 (4.9 to 33.1)	0.397
Epinephrine (pmol/L)	0.45 (0.09 to 0.90)	0.66 (0.28 to 1.20)	0.100
Maximal tumor diameter (cm) ^*b,c*^	5.3 ± 2.2	6.7 ± 3.2	**0.012**
Paraganglioma ^*b*^	53 (18.7%)	16 (37.2%)	**0.005**
Preoperative antihypertensives			
α-AR antagonist ^*d*^	246 (86.6%)	37 (86.0%)	0.918
Selective α1-AR antagonist	144 (58.5%)	20 (54.1%)	0.607
β-AR antagonist	65 (22.9%)	12 (27.9%)	0.470
Calcium channel blocker	73 (25.7%)	20 (46.5%)	**0.005**
Combined ^*b*^	83 (29.2%)	22 (51.2%)	**0.004**
Intravenous fluid	140 (49.6%)	22 (51.2%)	0.853
Preoperative SBP (mmHg)	125 ± 17	131 ± 13	**0.040**
Preoperative DBP (mmHg)	77 ± 12	81 ± 10	**0.025**
Preoperative HR (bpm)	74 ± 11	78 ± 9	**0.020**
Period of surgery ^*b*^			**0.005**
2005–2009	65 (22.9%)	7 (16.3%)	
2010–2013	61 (21.5%)	19 (44.2%)	
2014–2017	158 (55.6%)	17 (39.5%)	
Duration of anesthesia (min)	214 ± 82	308 ± 148	**< 0.001**
Type of anesthesia ^*b*^			0.055
General	188 (66.2%)	22 (51.2%)	
Combined epidural-general ^*e*^	96 (33.8%)	21 (48.8%)	
Duration of surgery (min) ^*b*^	129 ± 75	223 ± 148	**< 0.001**
Type of surgery ^*b*^			**0.015**
Open	89 (31.3%)	23 (53.5%)	
Laparoscopic	192 (67.6%)	20 (46.5%)	
Transurethral	3 (1.1%)	0 (0.0%)	
Estimated blood loss (ml)	100 (50 to 300)	500 (100 to 1800)	**< 0.001**
Minimal hemoglobin (g/L)	110 ± 22	96 ± 22	**0.001**
Blood transfusion ^*b,f*^	37 (13.0%)	20 (46.5%)	**< 0.001**
Positive fluid balance (ml)	2200 (1500 to 3025)	3150 (2350 to 5475)	**0.001**
Use of antihypertensives	243 (85.6%)	41 (95.3%)	0.077
Combined antihypertensives ^*b,g*^	173 (60.9%)	34 (79.1%)	**0.021**
Use of vasopressors ^*b*^	129 (45.4%)	33 (76.7%)	**< 0.001**
Combined vasopressors ^*h*^	46 (16.2%)	16 (37.2%)	**0.001**

Data are presented as mean ± SD, number (%), or median (interquartile range)

*BMI* Body mass index, *ASA* American Society of Anesthesiologists, *AR* Adrenergic receptor, *SBP* Systolic blood pressure, *DBP* Diastolic blood pressure, *HR* Heart rate

^*a*^ Comparison between patients with or without postoperative complications

^*b*^ Variables adjusted in the multivariate model

^*c*^ Confirmed by postoperative pathologic examination results

^*d*^ Including phenoxybenzamine, doxazosin and terazosin. Forty four patients did not receive α-AR antagonist therapy due to normal blood pressure and serum catecholamine level before surgery. Diagnosis of pheochromocytoma was confirmed by postoperative pathologic examination

^*e*^ These patients also received postoperative patient-controlled epidural analgesia (PCEA)

^*f*^ Includes packed red blood cell, fresh frozen plasma, and/or concentrated platelet

^*g*^ Combined use of two or more intravenous antihypertensive drugs, including phentolamine, urapidil, nicardipine and/or esmolol

^*h*^ Combined use of two or more intravenous vasopressors, including phenylephrine, norepinephrine, and/or epinephrine

During the postoperative period, patients who developed postoperative complications received more vasopressors, were admitted to the ICU more frequently, underwent more and longer mechanical ventilation, and stayed longer in the ICU and hospital after surgery. No patient died during hospital stay (Table 3).

**Table 3:** Postoperative outcomes

Variables	Without postoperative complications (n = 284)	With postoperative complications (n = 43)	*P* value
Acute kidney injury ^*a*^	–	23 (53.5%)	–
Stage 1	–	19 (44.2%)	–
Stage 2	–	4 (9.3%)	–
Other postoperative complications	–	30 (69.8%)	–
Use of vasopressors ^*b*^	47 (16.5%)	19 (44.2%)	**< 0.001**
Duration of vasopressors (hr) ^*c*^	18.2 (11.8, 24.7)	22.1 (8.8, 35.4)	0.546
ICU admission	174 (61.3%)	41 (95.3%)	**< 0.001**
Use of MV	107 (37.7%)	33 (76.7%)	**< 0.001**
Duration of MV (hr) ^*d*^	4.1 (3.4, 4.8)	14.7 (5.6, 23.5)	**0.003**
Length of ICU stay (day) ^*e*^	1.4 (1.2, 1.5)	2.2 (1.8, 2.7)	**< 0.001**
Hospital stay after surgery (day)	5.9 (5.5, 6.2)	9.5 (7.5, 11.5)	**< 0.001**
In-hospital mortality	0 (0.0%)	0 (0.0%)	–

Data were presented as number of patients (percentage) or mean (95% confidence interval)

*ICU* Intensive care unit, *MV* Mechanical ventilation

^*a*^ Defined as increase in serum creatinine by ≥26.5 μmol/l within 48 h; or increase in serum creatinine to ≥1.5 times baseline, which is known or presumed to have occurred within the prior 7 days; or urine volume < 0.5 ml/kg/h for 6 h. Stage 1 was defined as serum creatinine 1.5–1.9 times baseline or ≥ 26.5 μmol/l increase or urine output < 0.5 ml/kg/h for 6–12 h; stage 2 was defined as serum creatinine 2.0–2.9 times baseline or urine output < 0.5 ml/kg/h for ≥12 h; stage 3 was defined as serum creatinine 3 times baseline or increase to ≥353.6 μmol/l or initiation of renal replacement therapy or urine output < 0.3 ml/kg/h ≥ 24 h or anuria ≥12 h

^*b*^ Requirement of vasopressors (norepinephrine or epinephrine) to maintain systolic blood pressure ≥ 90 mmHg after surgery

^*c*^ Results of patients who required vasopressors after surgery

^*d*^ Results of patients who required mechanical ventilation in the ICU after surgery

^*e*^ Results of patients who were admitted to the ICU after surgery

### Intraoperative blood pressure

**Fig. 2:**
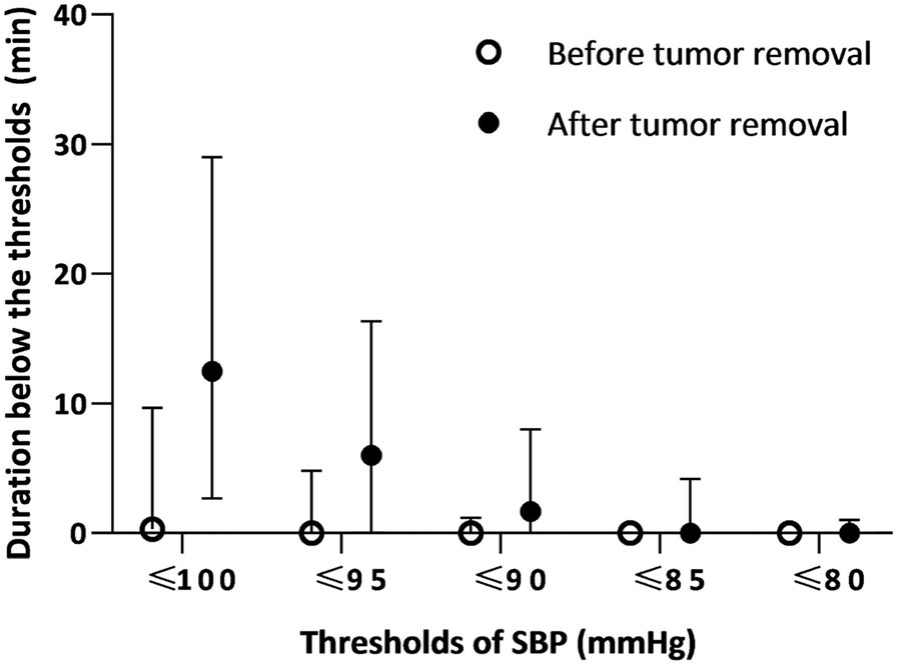
The duration of SBP below different thresholds before and after tumor removal. The spots and whiskers plots show medians (interquartile range). *P* < 0.001 for all thresholds of SBP. *P* < 0.01 (0.05/5) were considered statistically significant after Bonferroni correction. SBP, systolic blood pressure

The median percentage of no blood pressure record, calculated as the cumulated duration of no blood pressure record divided by the total duration of anesthesia, was 1.10% (interquartile range [IQR] 0.74 to 1.68%). The median percentage of blood pressure with artifacts, calculated as the cumulated duration of blood pressure with artifacts divided by the total duration of anesthesia, was 0.27% (IQR 0.00 to 0.65%). The durations of SBP below different thresholds were significantly longer after tumor removal than before (Fig. 2).

### Identification of potential confounding factors

Univariate analyses identified 24 variables with *P* values < 0.10 (Additional file 1: Supplementary Table 1). After testing for correlation, 12 variables were entered into the multivariate model to adjust for confounding factors, including male gender, ASA classification (3 + 4 vs. 1 + 2), maximal tumor diameter (cm), paraganglioma, preoperative combined antihypertensives, period of surgery, type of anesthesia (combined epidural-general vs. general), duration of surgery (minute), type of surgery (open vs. laparoscopic/transurethral), intraoperative blood transfusion, combined antihypertensives during surgery and use of vasopressor during surgery (Table 4).

**Table 4:** Association between intraoperative hyper−/hypotension and postoperative complications

Thresholds and durations	N	Univariate analysis		Multivariate analysis ^*a*^	
		OR (95% CI)	*P* value	OR (adjusted CI)	*P* value
SBP ≥200 mmHg				(95% CI), *P* < 0.05	
≥ 1 min	97	1.485 (0.760–2.902)	0.247	1.094 (0.497–2.407)	0.824
SBP ≥180 mmHg				(98.3% CI), *P* < 0.017 (0.05/3) ^*b*^	
≥ 1 min	186	1.185 (0.616–2.281)	0.611	0.735 (0.275–1.966)	0.455
≥ 5 min	99	1.130 (0.569–2.246)	0.727	0.719 (0.268–1.930)	0.425
≥ 10 min	63	1.321 (0.613–2.847)	0.478	0.792 (0.259–2.424)	0.619
SBP ≥160 mmHg				(99% CI), *P* < 0.01 (0.05/5) ^*b*^	
≥ 1 min	270	1.099 (0.463–2.611)	0.831	0.753 (0.175–3.240)	0.617
≥ 5 min	213	1.655 (0.801–3.423)	0.174	0.963 (0.308–3.016)	0.933
≥ 10 min	161	1.217 (0.640–2.314)	0.550	0.702 (0.254–1.940)	0.370
≥ 20 min	92	2.043 (1.055–3.958)	**0.034**	1.142 (0.406–3.213)	0.740
≥ 30 min	53	2.294 (1.090–4.828)	**0.029**	1.234 (0.387–3.933)	0.641
SBP ≤100 mmHg				(99.3% CI), *P* < 0.007 (0.05/7) ^*b*^	
≥ 1 min	278	4.065 (0.950–17.390)	0.059	3.141 (0.338–29.226)	0.166
≥ 5 min	243	2.335 (0.948–5.749)	0.065	1.710 (0.416–7.037)	0.306
≥ 10 min	211	2.685 (1.201–6.002)	**0.016**	1.839 (0.511–6.617)	0.200
≥ 20 min	162	3.017 (1.489–6.113)	**0.002**	1.809 (0.573–5.714)	0.164
≥ 30 min	121	3.043 (1.547–5.882)	**0.001**	1.871 (0.603–5.801)	0.136
≥ 40 min	83	4.293 (2.211–8.336)	**< 0.001**	2.535 (0.796–8.076)	0.030
≥ 50 min	58	4.392 (2.199–8.774)	**< 0.001**	2.625 (0.828–8.320)	0.024
SBP ≤95 mmHg				(99% CI), *P* < 0.01 (0.05/5) ^*b*^	
≥ 1 min	245	2.253 (0.915–5.551)	0.077	1.746 (0.446–6.834)	0.293
≥ 5 min	217	2.452 (1.096–5.487)	**0.029**	1.807 (0.528–6.180)	0.215
≥ 10 min	170	2.695 (1.330–5.458)	**0.006**	1.916 (0.640–5.735)	0.126
≥ 20 min	106	3.900 (2.008–7.547)	**< 0.001**	3.211 (1.081–9.536)	**0.006**
≥ 30 min	63	4.874 (2.465–9.634)	**< 0.001**	3.173 (1.012–9.950)	**0.009**
SBP ≤90 mmHg				(98.8% CI), *P* < 0.012 (0.05/4) ^*b*^	
≥ 1 min	207	2.424 (1.120–5.248)	**0.025**	1.653 (0.510–5.358)	0.283
≥ 5 min	147	2.588 (1.324–5.058)	**0.005**	1.662 (0.579–4.769)	0.226
≥ 10 min	101	3.396 (1.762–6.546)	**< 0.001**	2.160 (0.757–6.159)	0.065
≥ 20 min	53	5.122 (2.540–10.332)	**< 0.001**	3.680 (1.107–12.240)	**0.006**
SBP ≤85 mmHg				(98.3% CI), *P* < 0.017 (0.05/3) ^*b*^	
≥ 1 min	153	3.021 (1.512–6.033)	**0.002**	1.855 (0.647–5.317)	0.161
≥ 5 min	93	3.516 (1.822–6.783)	**< 0.001**	2.039 (0.739–5.621)	0.094
≥ 10 min	60	4.692 (2.361–9.325)	**< 0.001**	3.975 (1.321–11.961)	**0.003**
SBP ≤80 mmHg				(95% CI), *P* < 0.05	
≥ 1 min	98	5.062 (2.580–9.935)	**< 0.001**	3.465 (1.484–8.093)	**0.004**

*N* Number, *SBP* Systolic blood pressure, *OR* Odds ratio, *CI* Confidence interval

^*a*^ Independent factors with *P* values < 0.10 in univariate analyses or were considered clinically important were included in the multivariate logistic regression model. These included male gender, ASA classification (3 + 4 vs. 1 + 2), maximal tumor diameter (cm), paraganglioma, preoperative combined antihypertensives, period of surgery, type of anesthesia (combined epidural-general vs. general), duration of surgery (min), type of surgery (open vs. laparoscopic/transurethral), intraoperative blood transfusion, combined antihypertensives during surgery and use of vasopressors during surgery. History of diabetes mellitus and previous stroke were excluded due to correlation with ASA classification; preoperative calcium channel blocker, SBP, DBP and HR were excluded due to correlation with preoperative combined antihypertensives; duration of anesthesia and intraoperative positive fluid balance were excluded due to correlation with duration of surgery; intraoperative minimal hemoglobin and estimated blood loss were excluded due to correlation with intraoperative blood transfusion; use of antihypertensives during surgery was excluded due to correlation with combined antihypertensives during surgery; use of combined vasopressors during surgery were excluded due to correlation with use of vasopressors during surgery

^*b*^ The threshold level of significance was corrected using the Bonferroni method

### Association between intraoperative hyper−/hypotension and postoperative complications

Multivariate Logistic regression analyses showed that SBP of ≤95 mmHg for ≥20 min (OR 3.211; 99% CI 1.081–9.536; *P* = 0.006), SBP of ≤90 mmHg for ≥20 min (OR 3.680; 98.8% CI 1.107–12.240; *P* = 0.006), SBP of ≤85 mmHg for ≥10 min (OR 3.975; 98.3% CI 1.321–11.961; *P* = 0.003), and SBP of ≤80 mmHg for ≥1 min (OR 3.465; 95% CI 1.484–8.093; *P* = 0.004) were significantly associated with an increased risks of postoperative complications after Bonferroni correction. On the other hand, intraoperative hypertension, defined as SBP of different thresholds for different durations, was not significantly associated with the development of postoperative complications (Table 4).

## Discussion

Results of this retrospective study showed that, in patients undergoing surgery for pheochromocytoma or paraganglioma, intraoperative hypotension was associated with an increased risk of postoperative complications; whereas intraoperative hypertension was not. The harmful effects of intraoperative hypotension seemed “dose-dependent”, i.e., higher threshold with longer duration or lower threshold with shorter duration was associated with an increased risk of postoperative complications. Our results provided further evidence regarding the blood pressure management for these patients during surgery.

To avoid ambiguity, postoperative complications and their severities were clearly defined in the present study. In our patients, 13.1% developed postoperative complications; this was consistent with the multicenter study of Brunaud et al. [9] who reported an incidence of 16% after laparoscopic adrenalectomy for pheochromocytoma. Among all individual complications, AKI was the most common one and occurred in 7.0% of our patients, higher than the reported rate of 2.8% after surgery for non-functional adrenal tumors [23], possibly due to more severe/frequent intraoperative hypotension [12, 24]. Other individual complications included hypoglycemia (2.8%), pulmonary infection (1.2%), and so forth (Table 1).

Cumulative evidence showed an association between intraoperative hypotension and postoperative morbidity in patients undergoing abdominal [25, 26], vascular [27] and cardiac surgeries [28]. Intraoperative hypotension was found to be related to the development of myocardial injury [12, 13, 26, 27, 29], AKI [11, 12, 29, 30], and even death [16, 17, 31] after surgery. However, evidence in patients undergoing surgery for pheochromocytoma or paraganglioma are limited. In line with the above results, our study found that intraoperative hypotension is an independent risk factor of postoperative complications. Gaujoux et al. [32] also reported that perioperative hemodynamic instability, defined as the need for a cumulative dose of norepinephrine of > 5 mg, is significantly associated with morbidity development after surgery for pheochromocytoma. Currently, there are no widely accepted definition of intraoperative hypotension. In a recent systematic review, elevated risks of end-organ injury were presented for prolonged exposure (≥10 min) to MAP < 80 mmHg, for shorter durations < 70 mmHg, and for any exposure < 55–50 mmHg [33]. In accordance with this trend, our study revealed that the risk of postoperative complications increased when SBP ≤95 mmHg for ≥20 min, SBP ≤90 mmHg for ≥20 min, SBP ≤85 mmHg for ≥10 min, and SBP ≤80 mmHg for ≥1 min (Table 4). However, it should be noted that our results do not demonstrate a causal relationship due to the retrospective study design. Furthermore, the external validity of the above thresholds and durations should consider treatment factors, such as fluid infusion and use of vasopressors.

The possible mechanisms underlying the development of end organ injuries from intraoperative hypotension may include the following. First of all, hypotension contributed to the imbalance of oxygen delivery-consumption in vital organs by decreasing oxygen supply [34]; the resulting ischemia-reperfusion injury then triggered inflammation responses which might be involved in the damage process [12, 17, 35]. In addition, intraoperative hypotension might be a marker of other intraoperative events and comorbidities that were associated with an increased risk of postoperative complications [36]. At last, improper use of catecholamine infusion during hypotension might have adverse effects on splanchnic blood flow which led to potentially deleterious consequences [37, 38].

Interestingly, our study did not find any significant association between intraoperative hypertension and postoperative complications. Similar results were also reported by others. For example, in a retrospective cohort study, Monk et al. [19] found that intraoperative hypotension, but not hypertension, was associated with increased 30-day mortality after non-cardiac surgery. One possible explanation is that patients with intraoperative hypertension often had experienced (and, thus, adapted to) hypertension before surgery; these might have made them more tolerable to intraoperative hypertension, but more vulnerable to hypotension. However, due to the retrospective nature of our study and the limited number of patients, the potential harmful effects of intraoperative hypertension cannot be excluded. Further studies are required to clarify whether and to what extent intraoperative hypertension affects outcomes in patients undergoing surgery for pheochromocytoma or paraganglioma.

In addition to the retrospective nature, there were some other limitations in our study. Firstly, given the long duration of this study, many innovations or new treatments were introduced during the studied period and might confound the results. However, after adjusting for period of surgery in the multivariate Logistic model, intraoperative hypotension remained an independent risk factor of postoperative complications (Table 4). Secondly, pheochromocytoma or paraganglioma are relatively rare diseases. Although the sample size was large in this study when compared with others, it was not enough to do further sensitivity analysis; for example, the impacts of hypotension with separate thresholds (SBP 95–90, 90–85, and 85–80 mmHg) for different durations (1–5, 5–10, and 10–20 min) on postoperative complications. Finally, patients’ data were collected until hospital discharge. In a prospective study, Woodfield et al. [39] found that about one-third of complications occurred between hospital discharge and 30 days after surgery. Our results might have underestimated the incidence of postoperative complications.

## Conclusions

For patients undergoing surgery for pheochromocytoma or paraganglioma, intraoperative hypotension is associated with increased postoperative complications; and the harmful effects of intraoperative hypotension are level- and duration-dependent. The effects of intraoperative hypertension need to be studied further.

## Supplementary information

**Additional file 1 Supplementary Table 1.** Univariate association between baseline/intraoperative variables and postoperative complications.

## Data Availability

The datasets used and/or analyzed during the current study are available from the corresponding author on reasonable request.

## Abbreviations

AKI: Acute kidney injury
AR: Adrenergic receptor
ASA: American Society of Anesthesiologists
BMI: Body mass index
CI: Confidence interval
DBP: Diastolic blood pressure
HR: Heart rate
ICU: Intensive care unit
IQR: Interquartile range
KDIGO: Kidney Disease Improving Global Outcomes
MAP: Mean arterial pressure
MV: Mechanical ventilation
OR: Odds ratio
PCEA: Patient-controlled epidural analgesia
SBP: Systolic blood pressure
