# Supplementary material for: Intraoperative hypotension is associated with increased postoperative complications in patients undergoing surgery for pheochromocytoma-paraganglioma: a retrospective cohort study
Source: BMC Anesthesiol. 2020 Jun 12;20:147. doi: 10.1186/s12871-020-01066-y (PMC7291712; doi:10.1186/s12871-020-01066-y)
Supplement: Supplementary file 1 — Additional file 1 Supplementary Table 1. Univariate association between baseline/intraoperative variables and postoperative complications. [file 12871_2020_1066_MOESM1_ESM.docx]

**Supplementary Table 1. Univariate association between baseline/intraoperative variables and postoperative complications**

| Variables | All patients (n=327) | | |
| --- | --- | --- | --- |
|  | N (missing data) | OR (95% CI) *^a^* | P value |
| Age (years) | 327 (0) | 1.009 (0.988-1.031) | 0.378 |
| Male gender | 147 (0) | 2.588 (1.324-5.058) | **0.005** |
| BMI (kg/m^2^) | 327 (0) | 0.989 (0.901-1.085) | 0.810 |
| Preoperative comorbidity |  |  |  |
| Diabetes mellitus | 50 (0) | 2.506 (1.185-5.299) | **0.016** |
| Coronary heart disease | 19 (0) | 0.352 (0.046-2.705) | 0.316 |
| Previous stroke | 16 (0) | 5.941 (2.085-16.928) | **0.001** |
| ASA classification (3+4 vs. 1+2) | 327 (0) | 2.000 (1.004-3.983) | **0.049** |
| Preoperative examination |  |  |  |
| Hemoglobin (g/L) | 315 (12) | 0.998 (0.981-1.016) | 0.850 |
| Serum catecholamine |  |  |  |
| Dopamine (pmol/L) | 146 (181) | 0.996 (0.965-1.029) | 0.810 |
| Norepinephrine (pmol/L) | 180 (147) | 0.999 (0.995-1.004) | 0.800 |
| Epinephrine (pmol/L) | 175 (152) | 1.035 (0.960-1.115) | 0.372 |
| Maximal tumor diameter (cm) *^b^* | 325 (2) | 1.223 (1.084-1.381) | **0.001** |
| Paraganglioma | 69 (0) | 2.583 (1.300-5.132) | **0.007** |
| Preoperative antihypertensives |  |  |  |
| α-AR antagonist *^c^* | 283 (0) | 0.953 (0.377-2.409) | 0.918 |
| Selective α1-AR antagonist | 164 (0) | 0.833 (0.416-1.669) | 0.607 |
| β-AR antagonist | 77 (0) | 1.304 (0.634-2.684) | 0.471 |
| Calcium channel blocker | 93 (0) | 2.513 (1.305-4.842) | **0.006** |
| Combined | 105 (0) | 2.537 (1.324-4.861) | **0.005** |
| Preoperative intravenous fluid | 162 (2) | 1.063 (0.559-2.019) | 0.853 |
| Preoperative SBP (mmHg) | 327 (0) | 1.023 (1.001-1.044) | **0.036** |
| Preoperative DBP (mmHg) | 327 (0) | 1.033 (1.004-1.063) | **0.023** |
| Preoperative HR (bpm) | 326 (1) | 1.060 (1.015-1.107) | **0.008** |
| Period of surgery |  |  |  |
| 2005-2009 | 72 (0) | Ref. |  |
| 2010-2013 | 80 (0) | 2.892 (1.136-7.363) | **0.026** |
| 2014-2017 | 175 (0) | 1.000 (0.629-1.588) | 0.998 |
| Duration of anesthesia (min) | 327 (0) | 1.008 (1.005-1.011) | **<0.001** |
| Type of anesthesia (combined epidural-general vs. general) *^d^* | 327 (0) | 1.869 (0.979-3.568) | **0.058** |
| Duration of surgery (min) | 327 (0) | 1.008 (1.005-1.012) | **<0.001** |
| Type of surgery (open vs. laparoscopic/transurethral) | 327 (0) | 2.520 (1.316-4.824) | **0.005** |
| Intraoperative minimal hemoglobin (g/L) | 237 (90) | 0.756 (0.639-0.894) | **0.001** |
| Intraoperative management |  |  |  |
| Estimated blood loss (100 ml) | 325 (2) | 1.053 (1.024-1.084) | **<0.001** |
| Blood transfusion *^e^* | 57 (0) | 5.805 (2.907-11.593) | **<0.001** |
| Positive fluid balance (100 ml) | 326 (1) | 1.044 (1.025-1.064) | **<0.001** |
| Use of antihypertensives | 284 (0) | 3.459 (0.805-14.855) | **0.095** |
| Combined antihypertensives *^f^* | 207 (0) | 2.424 (1.120-5.248) | **0.025** |
| Use of vasopressors | 162 (0) | 3.965 (1.882-8.353) | **<0.001** |
| Combined vasopressors *^g^* | 62 (0) | 3.066 (1.531-6.139) | **0.002** |

OR, odds ratio; CI, confidence interval; BMI, body mass index; ASA, American Society of Anesthesiologists; SBP, systolic blood pressure; DBP, diastolic blood pressure; HR, heart rate; ICU, intensive care unit; MV, mechanical ventilation. P values in bold indicate those <0.10.

*^a^* Calculated as patients with postoperative complications vs. those without.

*^b^* Confirmed by postoperative pathologic examination results.

*^c^* Including phenoxybenzamine, doxazosin and terazosin. 44 patients did not receive α-AR antagonist therapy due to normal blood pressure and serum catecholamine level before surgery. Diagnosis of pheochromocytoma or paraganglioma was confirmed by postoperative pathologic examination.

*^d^* These patients received postoperative patient-controlled epidural analgesia.

*^e^* Includes packed red blood cell, fresh frozen plasma and concentrated platelet.

*^f^* Combined use of two or more intravenous antihypertensive drugs, including phentolamine, urapidil, nicardipine and/or esmolol.

*^g^* Combined use of two or more intravenous vasopressors, including phenylephrine, norepinephrine, and/or epinephrine.
